# Decreased islet amyloid polypeptide staining in the islets of insulinoma patients

**DOI:** 10.1080/19382014.2024.2379650

**Published:** 2024-07-19

**Authors:** Chisaki Ishibashi, Sho Yoneda, Yukari Fujita, Shingo Fujita, Kento Mitsushio, Harutoshi Ozawa, Megu Y Baden, Takao Nammo, Junji Kozawa, Hidetoshi Eguchi, Iichiro Shimomura

**Affiliations:** a Department of Metabolic Medicine, Graduate School of Medicine, Osaka University, Suita, Japan; b Yoneda Clinic, Osaka, Japan; c Department of Lifestyle Medicine, Graduate School of Medicine, Osaka University, Suita, Japan; d Department of Diabetes Care Medicine, Graduate School of Medicine, Osaka University, Suita, Japan; e Department of Gastroenterological Surgery, Graduate School of Medicine, Osaka University, Suita, Japan

**Keywords:** Amylin, hypoglycemia, IAPP, insulinoma, islet, obesity

## Abstract

Islet amyloid polypeptide (IAPP) is a factor that regulates food intake and is secreted from both pancreatic islets and insulinoma cells. Here, we aimed to evaluate IAPP immunohistochemically in islets or insulinoma cells in association with clinical characteristics. We recruited six insulinoma patients and six body mass index-matched control patients with pancreatic diseases other than insulinoma whose glucose tolerance was confirmed to be normal preoperatively. IAPP and IAPP-insulin double staining were performed on pancreatic surgical specimens. We observed that the IAPP staining level and percentage of IAPP-positive beta cells tended to be lower (*p* = 0.1699) in the islets of insulinoma patients than in those of control patients, which might represent a novel IAPP expression pattern under persistent hyperinsulinemia and hypoglycemia.

Islet amyloid polypeptide (IAPP), also known as amylin, is a hormone that is secreted mainly by pancreatic beta cells and regulates food intake and glucose metabolism.^1,2^ IAPP suppresses glucagon secretion, inhibits gastric emptying, and reduces food intake via the hypothalamus.^3,4^ IAPP secretion is regulated by many factors. Circulating IAPP concentrations are increased in individuals with obesity,^5,6^ hypertension,^7,8^ and renal failure.^9,10^ It is also increased by the injection of glucose,^11–13^ arginine,^14^ fatty acids,^15^ and dexamethasone.^16^ Regarding the diabetic state, basal secretion of IAPP is maintained in non-insulin-dependent type 2 diabetics but is decreased by glucose stimulation,^5,6^ while basal and glucose-stimulated secretion are markedly decreased in type 1 diabetes, an insulin-dependent state.^17,18^ IAPP is also secreted by insulinoma cells.^19,20^ However, the IAPP staining level in insulinoma patients characterized by persistent hyperinsulinemia and hypoglycemic episodes has not been investigated in detail thus far. We previously showed that the expression levels of glucose transporter 2 (GLUT2) and glucagon-like peptide 1 (GLP-1) receptor in beta cells in nontumor areas were downregulated by hyperinsulinemia.^21^ Similarly, we hypothesized that there might be a mechanism regulating IAPP expression in insulinoma patients. In this study, we immunohistochemically evaluated pancreatic IAPP staining in six insulinoma patients and six control patients to enhance our understanding of IAPP regulation in persistent hyperinsulinemia and hypoglycemia.

We evaluated six insulinoma patients who underwent pancreatectomy at the Department of Gastroenterological Surgery, Osaka University Hospital, between 2007 and 2021. We evaluated six control patients who underwent pancreatectomy for pancreatic cancer or cystic disease between 2007 and 2011 and were confirmed to have normal glucose tolerance by a 75-g oral glucose tolerance test (OGTT) performed preoperatively. The control patients were diagnosed with normal glucose tolerance (NGT) after meeting the criteria of both fasting plasma glucose levels less than 6.1 mmol/L and 2 h-OGTT glucose levels less than 7.8 mmol/L. Regarding clinical information, we obtained data on age, sex, height, body weight at the time of operation, and the levels of HbA1c, fasting plasma glucose and fasting plasma insulin from the medical records. To convert HbA1c (Japan Diabetes Society) to HbA1c (National Glycohemoglobin Standardization Program) values, the following equation was used: National Glycohemoglobin Standardization Program (percentage) = 1.02 × Japan Diabetes Society (percentage) + 0.25%.^22^ Information on the duration of the disease (from the time of diagnosis to operation) was also obtained for insulinoma patients. None of the insulinoma patients had evidence of impaired glucose tolerance or diabetes preoperatively.

The surgical samples were fixed in formaldehyde and embedded in paraffin immediately after surgery. The paraffin blocks were cut into 5-µm-thick sections. Among the six insulinoma patients, four underwent distal pancreatectomy, one underwent middle pancreatectomy, and one underwent enucleation of the insulinoma. We investigated sections from a block that contained both tumor and nontumor areas from each patient who underwent distal or middle pancreatectomy and sections from a block with only a tumor area in a patient who underwent enucleation of an insulinoma. In addition, we studied sections from a block of normal pancreatic tissue attached to pathological lesions resected by surgery in control patients. All sections from control patients were confirmed to be normal, without inflammation or fibrosis by hematoxylin and eosin staining. Each subsequent histological analysis was performed on one pancreatic section from each patient. We repeated the staining at least three times for all patients, and we confirmed that similar staining results could be obtained.

The primary and secondary antibodies and chromogenic substrates used in this study are listed in the Supplementary Tables. Sections were deparaffinized in xylene and rehydrated in ethanol, followed by heat-mediated epitope retrieval with pH 6 citrate buffer (Code No.: S1700; DAKO Japan, Kyoto, Japan). To evaluate the intensity of IAPP staining, we blocked endogenous peroxidase activity by the addition of 1% H2O2 in methanol for 20 min and incubated the sections in blocking buffer containing 3% bovine serum albumin for 30 min. Then, the sections were incubated overnight in rabbit anti-IAPP immunoglobulin as the primary antibody diluted at 1:800, as previously described.^19^ The next day, the sections were washed and incubated with biotinylated secondary goat immunoglobulin for 1 h at room temperature. Antibody binding was amplified by an avidin – biotin complex and detected by diaminobenzidine tetrahydrochloride. Sections were counterstained with hematoxylin. To evaluate the ratio of beta cells costained with IAPP, we performed double-immunofluorescence staining for IAPP and insulin. First, the sections were incubated overnight with anti-IAPP immunoglobulin as the primary antibody, as described above, and incubated with anti-rabbit biotinylated immunoglobulin as the secondary antibody, followed by incubation with Alexa 488-conjugated streptavidin. Then, the sections were incubated with guinea pig anti-insulin immunoglobulin as the primary antibody and with anti-guinea pig Alexa 555-conjugated immunoglobulin as the secondary antibody. Sections were counterstained with 4,’6-diamidino-2-phenylindole (DAPI). For evaluation of the percentages of amylin-positive beta cells, we examined a randomly chosen field at high magnification (20× objective). We analyzed 9 (6.5–10) islets for each insulinoma case and 8.5 (4.5–12) islets for each NGT case. We counted insulin-positive cells shown in red as beta cells. Since IAPP stains green and cells double positive for insulin and IAPP are shown in yellow in merged images, we counted cells shown in yellow in merged images as IAPP-positive beta cells. Conversely, cells that showed red in merged images were considered as IAPP-negative beta cells. The percentages of IAPP-positive beta cells were calculated by dividing the number of IAPP-positive beta cells by the number of beta cells.

All statistical analyses were performed with JMP Pro 15 software (SAS Institute Inc., Cary, NC, USA). Values are presented as median (first quartile-third quartile). Differences in the number of IAPP-positive cells/beta cells were analyzed by means of the Wilcoxon test. *p* < 0.05 was considered to indicate statistical significance.

This study was performed in accordance with the principles of the Declaration of Helsinki. Approval was granted by the Osaka University Research Ethics Committee (approval number: 17419–3). We obtained written informed consent from all the participants in the study. The study was announced on the website of our department at Osaka University Hospital, and all the patients were allowed to refuse to participate in the study.

The clinical characteristics of the insulinoma patients are presented in Table 1. The patients are listed in ascending order according to their BMI value. Insulinoma patients 1–4 were nonobese with a BMI of 20.0–23.9 kg/m^2^, and insulinoma patients 5 and 6 were obese with a BMI of 29.6 and 30.7 kg/m^2^, respectively. We evaluated the severity of hypoglycemia in insulinoma patients by the most severe symptoms caused by hypoglycemia, the presence or absence of a history of hospital transportation due to hypoglycemic attack, and hypoglycemic episodes under 2.8 mmol/L within 3 days after hospitalization. Only two insulinoma patients (patients 2 and 5) had never experienced hypoglycemic coma. There was no obvious association between hypoglycemia severity and tumor size in insulinoma patients. During the follow-up period of 3–10 years after surgery, only one insulinoma patient (patient 5) developed diabetes. Insulinoma patient 5 had concomitant pancreatic cystic tumors and habits of overeating and snacking late at night. The clinical characteristics of the control NGT patients are listed in ascending order according to BMI in Table 2. There was no difference in age, sex, or BMI between the control and insulinoma groups. The underlying disease types that required surgical treatment were intraductal papillary mucinous neoplasm in three patients and pseudocysts, tubular adenocarcinomas, and simple cysts in one patient.

**Table 1.** Preoperative characteristics of insulinoma patients.

Pt	Age (yr)	Sex	BMI (kg/m)^2^	Symptoms	History of hospital transportation^a^	Hypoglycemic episodes during hospitalization^b^	HbA1c (%)	Glu (mM)	Ins (pM)	Tumor size (mm^2^)	Classification^c^	Surgical procedure	Postoperative diabetes^d^
1	38	F	20.0	Loss of consciousness	Yes	4	4.4	3.0	136	10	NET G1	CP	No
2	70	F	23.4	Abnormal behavior	No	3	4.9	2.3	94	10	NET G1	EN	No
3	63	F	23.5	Loss of consciousness	Yes	2	5.2	1.9	72	17	NET G2	DP	No
4	73	M	23.9	Loss of consciousness	Yes	6	4.4	2.7	48	18	NET G1	DP	No
5	47	F	29.6	Numbness of hands	No	3	4.8	3.7	170	15	NET G1	DP	Yes
6	72	F	30.7	Disturbance of consciousness	Yes	3	4.5	2.6	90	9	NET G1	DP	No

Abbreviations: Pt, patient; BMI, body mass index; Glu, glucose; Ins, insulin; CP, central pancreatectomy; EN, enucleation; DP, distal pancreatectomy. ^a^History of hospital transportation due to hypoglycemic attack; ^b^Hypoglycemic episodes under 2.8 mM within 3 days after hospitalization; ^c^Pathological classification based on WHO classification of neuroendocrine neoplasms of 2010 or 2019 edition; ^d^Onset of diabetes after surgery during follow-up periods of 3–10 years.

**Table 2.** Preoperative characteristics of NGT patients.

Control patient	Age (yr)	Sex	BMI (kg/m)^2^	HbA1c (%)	Glucose (mM)	Insulin (pM)	Background disease	Surgical Procedure
1	72	F	19.9	5.3	4.7	36	IPMN	PD
2	50	F	21.4	5.5	4.9	36	Pseudocyst	DP
3	63	M	22.1	5.5	4.8	17	Tubular adenocarcinoma	PD
4	75	F	22.5	5.4	5.0	51	IPMN	DP
5	70	M	25.0	5.8	5.5	47	IPMN	PD
6	41	F	28.7	5.4	5.9	-	Simple cyst	DP

Abbreviations: NGT, normal glucose tolerance; BMI, body mass index; IPMN, intraductal papillary mucinous neoplasm; PD, pancreaticoduodenectomy; DP, distal pancreatectomy.

Figure 1 shows representative images of IAPP staining in nontumor area islets (A-E) and insulinoma cells (F-K) of insulinoma patients and in islets of control patients (L-Q). The images (from left to right) are shown in the order of patient numbers, from one to six. First, we focused on IAPP staining in islets in the nontumor area of insulinoma patients. The IAPP staining levels in islets in the nontumor area of insulinoma patients (Figure 1A-E) were weaker than that in the islets from the control patients (Figure 1L-Q). We detected especially low IAPP staining in islets, especially in patients 1 and 4 (Figure 1A and C respectively). In both patients, there was a history of hospital transportation due to hypoglycemic coma, and the preoperative HbA1c values were both 4.4%, which were lower than those of the other insulinoma patients. On the other hand, patients 3, 5, and 6, two of whom were obese, had moderate IAPP staining in the islets (Figure 1B, D and E, respectively). Next, we focused on the tumor area of insulinoma patients. Higher IAPP staining was observed in the insulinoma cells of patients 3 and 6 (Figure 1H and K, respectively) than in those of patients 1 and 4 (Figure 1F and I, respectively) or in patients 2 and 5 (Figure 1 G and J, respectively), both of whom had no history of hypoglycemic coma. No correlation was observed between the IAPP staining levels in the tumor area and the nontumor area. In addition, we performed double immunofluorescence staining for IAPP and insulin. A representative image of an islet from control NGT patient 2, who had the highest percentage of IAPP-positive beta cells, is shown in Figure 2A. Almost all the beta cells were stained with IAPP in control NGT patient 2. On the other hand, a representative image of an islet from insulinoma patient 4, who had the lowest percentage of IAPP-positive beta cells, is shown in Figure 2B. Only a few beta cells were stained with IAPP in insulinoma patient 4. Figure 2C shows the percentages of IAPP-positive beta cells in each patient sample. The percentage of IAPP-positive beta cells tended to be lower in the insulinoma group than in the NGT control group, although the difference was not significant (72 (30–84)% vs. 84 (75–93)%, *p* = 0.1699). Then, we evaluated the association between the percentage of IAPP-positive beta cells and each clinical parameter (Supplementary Figure). We found a significant association between the percentage of IAPP-positive beta cells in islets in nontumor areas and in the samples of insulinoma patients with hypoglycemic episodes (supplementary figure H).

**Figure 1.**
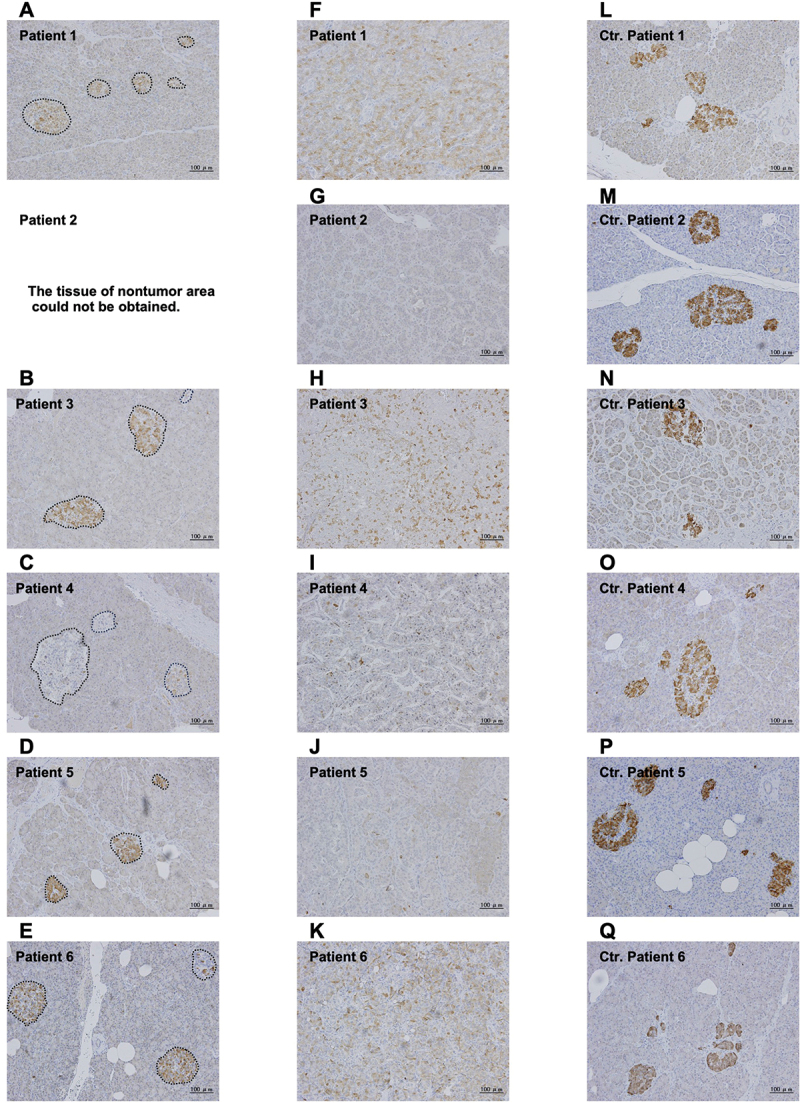
Representative images of IAPP staining of the nontumor area islets of insulinoma patients (A-E), the tumor area of insulinoma patients (F-K), and islets of control (Ctr) patients (L-Q). Patient 1: weak IAPP staining of islets (A, 49% IAPP-positive beta cells) and tumor area (F). Patient 2: hardly any staining of the tumor area (G). Tissue from the nontumor area in this patient could not be analyzed because of the operative enucleation procedure. Patient 3: moderately strong staining of islets (B, 72% IAPP-positive beta cells) and diffuse strong staining of the tumor area (H). Patient 4: weak and diffuse negative staining of the islets (C, 12% IAPP-positive beta cells) and sparse, weak staining of the tumor area (I). Patient 5: moderately strong staining of the islets (D, 82% IAPP-positive beta cells) and sparse staining of the tumor area (J). Patient 6: moderately strong staining of islets (E, 85% IAPP-positive beta cells) and diffuse strong staining in the tumor area (K). On the other hand, relatively strong IAPP staining was detected in the islets of all the control patients (L-Q). The percentages of IAPP-positive beta cells were as follows: L, 92%; M, 93%; N, 54%; O, 82%; P, 82%; and Q, 85%. We analyzed 9 (6.5–10) islets for each insulinoma case. Values are median (first quartile-third quartile).

**Figure 2.**
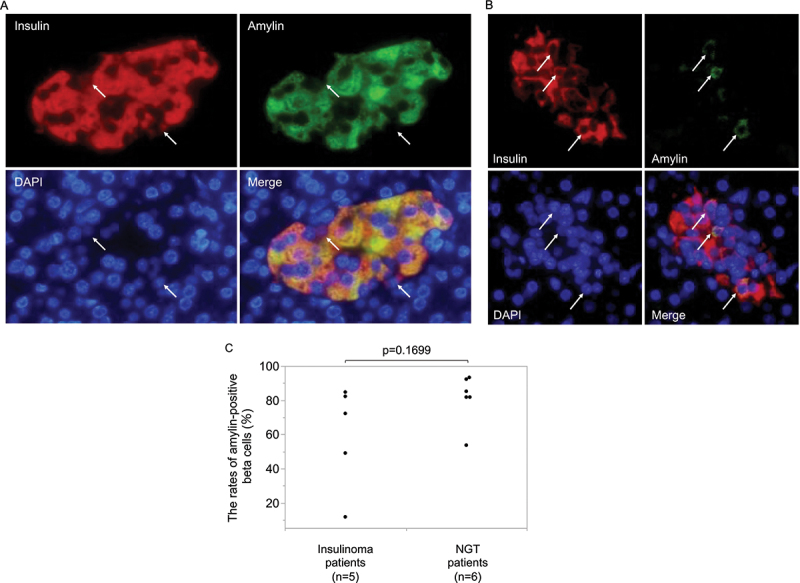
Representative images of islets from patients with the highest (control patient 2) and lowest (patient 4) percentages of IAPP-positive beta cells are shown in figure 2A and figure 2B, respectively. Most of the beta cells without white arrows costained with IAPP, as shown in figure 2A, and only a few beta cells with white arrows costained with IAPP, as shown in figure 2B. Then, we compared the percentages of IAPP-positive beta cells in the insulinoma group with those in the normal glucose tolerance (NGT) group (figure 2C). We found that they tended to be lower in the insulinoma group than in the NGT group (*p* = 0.1699). We analyzed 9 (6.5–10) islets for each insulinoma case and 8.5 (4.5–12) islets for each NGT case, respectively.

In this study, the IAPP staining levels in islets of the nontumor areas in the insulinoma patient samples tended to be lower than those in the control patient samples, and the staining level was especially low in the samples of patients who had experienced severe hypoglycemia. To our knowledge, this is the first report of immunohistochemical staining for IAPP in islets in the nontumor area of insulinoma patients compared with those of NGT individuals. These results suggest a novel mechanism by which IAPP expression is regulated in chronic hyperinsulinemia and hypoglycemia. In general, glucagon, catecholamines, growth hormone, and cortisol are the main counterregulators of hypoglycemia.^23,24^ Although the increase in IAPP in response to glucose loading.^11–13^ is well known, the regulation of IAPP release in relation to hypoglycemic conditions has rarely been studied. Among the limited existing studies, it was reported that in situ hybridization of IAPP RNA in rat islets was strikingly decreased after 12 days of insulin-induced hypoglycemia.^25^ Another study indicated that circulating IAPP was suppressed during insulin-induced hypoglycemia in nondiabetic human subjects.^26^ Furthermore, other previous studies have shown that IAPP-mediated regulation of gastric emptying,^27^ and food intake,^28^ are reduced under hypoglycemic conditions in rat models. In this study, we first found that the staining level of IAPP tended to be reduced immunohistochemically in the human islets of insulinoma patients, another IAPP-mediated mechanism for escaping hypoglycemia. The tendency for reduced IAPP protein levels might be due to decreased mRNA expression, as shown in a previous report.^25^ We previously revealed that the immunohistochemical expression levels of both GLUT2 and GLP-1 receptor were lower in the islets of insulinoma patients than in the islets of NGT patients and that exposure to high concentrations of insulin resulted in a decrease in both GLUT2 and GLP-1 receptor proteins in MIN6 cells in an in vitro study.^20^ Similarly, in this study, we speculate that persistent hyperinsulinemia and/or subsequent hypoglycemia may lead to a reduction in IAPP synthesis or accelerated degradation of IAPP in the islets of nontumor areas in insulinoma patients. In our study of the associations between the percentage of IAPP-positive beta cells and clinical parameters, the lower percentage of IAPP-positive beta cells was related to more hypoglycemic episodes (Supplementary Figure H). Although speculative, this low rate of IAPP-positive beta cells might be partially explained by the regulation for keeping petite to avoid hypoglycemia. Some previous reports have suggested that IAPP is suppressed in obese individuals;^5,6^ however, we found no significant correlation between BMI and the percentage of IAPP-positive beta cells (Supplementary Figure B), so whether IAPP staining levels in islets in insulinoma patients receive feedback from obesity is unclear.

This study has several limitations. First, we could not assess serum IAPP concentrations. IAPP acts on the hypothalamus through the blood‒brain barrier. It was not confirmed whether the amount of IAPP in pancreatic tissue contributed to circulating IAPP levels. In addition, we could not directly evaluate the intensity of IAPP staining levels in islets or insulinoma cells. To confirm our speculation on the association between the IAPP level and mechanisms for avoiding hypoglycemia, assessment of circulating IAPP and quantification of IAPP on pancreatic tissue are needed. Second, only a small number of patients were studied; thus, we cannot generalize the results in a strict sense. Third, we studied only IAPP, a hormone that regulates appetite, while other hormones, such as ghrelin, cholecystokinin, peptide YY, glucagon-like peptide, and oxyntomodulin,^29–31^ were not considered. Finally, in most insulinoma patients, it is difficult to obtain precise, accurate, and detailed information about the duration of the disease, degree of appetite, changes in body weight, and preoperative glucose tolerance.

In conclusion, we observed that immunohistochemically detected IAPP staining in the islets of insulinoma patients was decreased, which might represent a novel form of IAPP regulation under persistent hyperinsulinemia and hypoglycemia. This knowledge may contribute to a better understanding of the human pathophysiological response to hypoglycemic conditions.

## Supplementary Material

Supplementary fig.jpg

Supplementary Tables.docx
