# Supplementary material for: Decreased islet amyloid polypeptide staining in the islets of insulinoma patients
Source: Islets. 2024 Jul 19;16(1):2379650. doi: 10.1080/19382014.2024.2379650 (PMC11262209; doi:10.1080/19382014.2024.2379650)
Supplement: Supplementary Tables.docx [file KISL_A_2379650_SM2403.docx]

**Supplementary Table**

1. Primary antibodies

| Antigen | Species | Source | RRID | Dilution |
| --- | --- | --- | --- | --- |
| Amylin | Rabbit | BMA Biomedicals, Switzerland | AB_518720 | 1:800 |
| Insulin | Guinea pig | Dako Japan, Kyoto, Japan | AB_10013264 | 1:1000 |

1. Secondary antibodies

| Antigen | Species | Source | RRID | Dilution |
| --- | --- | --- | --- | --- |
| Rabbit | Goat (biotinylated) | Vector Laboratories, CA, USA | AB_2313606 | 1:200 |
| Guinea pig | Goat (Alexa Fluor 555-conjugated) | Thermo Fisher Scientific, MA, USA | AB_2535856 | 1:200 |

1. Chromogenic substrates

| Chromogenic substrates | Source | RRID | Dilution |
| --- | --- | --- | --- |
| Avidin-biotin complex kit | Vector Laboratories, CA, USA |  |  |
| 3,3-diaminobenzidine | Vector Laboratories, CA, USA |  |  |
| Streptavidin (Alexa Fluor 488-conjugated) | Molecular Probes, OR, USA | AB_2315383 | 1:200 |
